# Association of Urinary Bisphenols Concentration with Asthma in Korean Adolescents: Data from the Third Korean National Environmental Health Survey

**DOI:** 10.3390/toxics9110291

**Published:** 2021-11-04

**Authors:** Baek Kiook, Park Jong-Tae, Kwak Kyeongmin

**Affiliations:** Department of Occupational and Environmental Medicine, Korea University Ansan Hospital, Ansan 15355, Korea; bko8899@gmail.com (B.K.); impjt@korea.ac.kr (P.J.-T.)

**Keywords:** asthma, bisphenol, endocrine disruptor

## Abstract

The effects of bisphenol A (BPA) on asthma have been reported in various in vitro, animal, and human epidemiologic studies. However, epidemiological studies on the effects of bisphenol S (BPS) and bisphenol F (BPF), which are substitutes of BPA, on asthma are lacking. The purpose of this study was to identify the association between BPA, BPS, and BPF and asthma. An asthma-related questionnaire; urinary BPA, BPS, BPF; and the possible confounders were analyzed among 922 adolescents aged 12–17 years who participated in the Korean National Environmental Health Survey 2016. In males, urinary BPA, BPS, and BPF did not show a significant relationship with the lifetime prevalence of asthma. In females, urinary BPS was higher in the asthma group (*p* < 0.01). High urinary BPS showed a significant relationship with a high odds ratio (OR) of lifetime asthma prevalence in the model adjusted for possible confounders (*p* < 0.05). High urinary BPS was particularly associated with an increase in the OR of asthma diagnosed after the age of 60 months (*p* < 0.01). Urinary BPS was significantly associated with asthma diagnosis, especially after the age of 60 months, among Korean adolescent females.

## 1. Introduction

Bisphenols are widely used in plastic products, receipt ink, and medical equipment production [1,2]. Humans are likely exposed to bisphenols through contact with these products. Bisphenols act as endocrine disruptors in the body, causing various toxic effects such as endocrine dysfunction, cardiovascular disease, cancer, metabolic disease [3], reproductive system disorders, and affecting the immune system and oxidative stress [4]. In particular, the toxicity of bisphenol A (BPA) (4,4′-(propane-2,2-diyl) diphenol) is widely known [5], and there are restrictions on its use in industry, consumer products including epoxy resin-based paints, and thermal paper [6]. As a result, it has been replaced with other bisphenol analogues, such as bisphenol F (BPF) (4,4-dihydroxydiphenyl-methane) or bisphenol S (BPS) (4,4′-sulfonylbisphenol). However, the toxicity of bisphenol A analogues is expected to be similar to that of BPA as a result of similar chemical properties and has been reported in several studies [7,8].

Bisphenols have been shown to be toxic in individuals with allergic diseases. The immunotoxicity of BPA, which has been used for a long time, has been actively studied and reported. Animal and in vitro studies have shown that bisphenols increase IL-4 production by T helper (Th) 2 cells [9], increase Th1/Th2 response [10], and decrease CD4+ C25+ regulatory T cells [11]. The associations of BPA with serum, cord IgE [12,13], and the prevalence of asthma have been reported in human epidemiological studies [14,15,16].

BPS has similar chemical properties to BPA; however, it has high stability and a long biological half-life in animals and humans [17,18]. Both BPS and BPF have been reported to be toxic to the body as endocrine disruptors and to affect cytokine and chemokine secretion in the immune system [19]. However, human epidemiological studies on allergic diseases of bisphenol A analogues other than BPA are insufficient.

The Korean National Environmental Health Survey (KoNEHS) is being conducted by national institutions to identify diseases caused by environmental pollution in Korea. Samples from the Korean population have been collected since 2009 under the supervision of the National Institute of Environmental Research, and the results have been published with raw data [20]. In particular, in the third survey conducted after 2015, questionnaires and human sample analyses were conducted on children and adolescents. We attempted to report the relationship between urinary BPA, BPS, and BPF level, a biological marker of exposure to each bisphenols, and the prevalence of asthma using raw data from adolescents (middle and high school students) from the KoNEHS.

## 2. Materials and Methods

### 2.1. Study Population, Sampling, and Survey

The KoNEHS is a nationally representative population-based cross-sectional study that began in 2009 to assess current levels of exposure to environmental chemicals and several clinical markers, together with demographic and behavioral characteristics of the general Korean population. The survey was conducted among middle and high school students in 2016. Students were recruited throughout the country using multiple stage sampling method, following region, sex, and age stratification based on population data of Korea, and the educational institutions were used as sampling units [21]. In the KoNEHS 2016, 922 middle to high school students (aged 12–17 years) were recruited using a two-stage proportionally stratified sampling design based on sex, age, and geographical characteristics. Twenty-two patients with missing urinary BPA, BPS, and BPF samples were excluded from the analysis. Five females had missing values for serum IgE (one with asthma and four without); therefore, they were excluded from the correlation analysis of serum IgE and other variables.

The survey was conducted in the form of a written survey and one-on-one interviews with caregivers. For urine and blood samples, a medical institution adjacent to an educational institution was utilized. Subjects visited the medical institution, and body measurements and biological sample collections were performed by the medical staff. The blood sample was collected in a serum separation tube (SST), and after inversion mixing, it was allowed to stand for 30 min and then centrifuged at 3500 rpm to separate the serum. The urine samples were collected in a sterilized specimen cup and then shaded. Spot urine samples were transferred to the laboratory under cooling conditions with ice in an icebox and were stored at −20 °C before analysis. A detailed description of the collection and analysis of urine samples has been described previously [22,23].

### 2.2. Measurement of Urinary Bisphenols

BPA, BPS, and BPF levels were measured in urine samples. Urinary bisphenols were measured by ultra-performance liquid chromatography-tandem mass spectrometry (UPLC-MS/MS) equipped with electrospray ionization (ESI). A column (RP18-e) filled with C18 was used in a tube with an inner diameter of 3 mm and length of 100 mm.

The standard solutions for analysis were as follows: 99% BPA (2,2-bis(4-hydroxyphenyl) propane) (Cambridge, UK), 99% BPF (4,4′-dihydroxydiphenylmethane) (Sigma Aldrich, St.Louis, MO, USA), and 98% BPS (4,4′-sulfonyldiphenol) (Sigma Aldrich). Quality control procedures for all analytes were performed according to the National Institute of Environmental Research of Korea protocol. The method detection limits (MDLs) for BPA, BPS, and BPF were 0.075, 0.019, and 0.084 μg/L, respectively. The percentage of values below the detection limit among males were 13 (2.7%), 265 (55.4%), and 153 (32.0%) for urinary BPA, BPF, and BPS. The percentage of values below the detection limit among females were 11 (2.6%), 255 (60.4%), 173 (41.0%) for urinary BPA, BPF, and BPS, respectively.

### 2.3. Outcome Variables

As for the outcome variables, diagnosis of asthma and serum IgE was analyzed. In this study, information of ‘diagnosis of asthma by a doctor’ (yes/no), ‘the first diagnosis time of asthma’ (age < 6, 7–12, 13–18, 19–24, 25–36, 37–60, >60 months), and ‘current asthma treatment (yes/no)’ were collected by using the questionnaire. The first diagnosis time of asthma was re-categorized as 0–60 months and >60 months.

Serum IgE was measured with a photometer by a competitive immunoassay using chemiluminescent (ADVIA Centaur, Siemens, Munich, Germany) and IMMULITE 2000 XPi (Siemens medical Sol., Washington, DC, USA). The analytical range was 1.0–2000 mg/L.

### 2.4. Possible Confounders

Urinary cotinine level, which is a biomarker of tobacco smoke exposure [24], were collected as possible confounders that may affect asthma prevalence. Urinary cotinine levels were analyzed using gas chromatography-mass spectrometry. The column was 0.25, 250 µm, 30 m long, and an HP-5MS or equivalent column was used. Cotinine (1 mg/mL; C_10_H_12_N_2_O) (Sigma Aldrich) was used as the standard material. The MDL of cotinine was 0.3 µg/L. The percentage of values below the detection limit among males was 21 (4.4%) for urinary cotinine. The percentage of values below the detection limit among females was 33 (7.8%) for urinary cotinine, respectively.

Monthly income and age were extracted from the survey questionnaire as possible confounders.

### 2.5. Statistical Analysis

Urinary bisphenols and cotinine levels were standardized by dividing the urinary creatinine levels. In cases where the value was below the detection limit, the value was replaced by “MDL/√2”. Urinary bisphenols and cotinine had right-skewed distribution, therefore, a non-parametric method was used for univariate analysis, and natural log-transformation was performed for correlation and regression analysis.

Univariate analysis comparing “ever diagnosed with asthma” group and “never diagnosed with asthma” group was performed separately for each sex. Age; family income; body mass index (BMI); urinary BPA, BPS, BPF; cotinine; and serum IgE levels were presented for the “ever diagnosed with asthma” group and “never diagnosed with asthma” group. For continuous variables, the median, 25th percentile, and 75th percentile were presented, and for categorical variables, proportions were presented. For the *p*-value calculation, the Mann–Whitney U test was used for continuous variables, and the chi-square test was used for categorical variables. Correlations between continuous variables were calculated using Pearson’s correlation coefficients to calculate *p*-values considering the complex survey design, and the non-parametric sample-weighted bootstrapping procedure was used. Bootstrap resampling was performed 2000 times.

Logistic regression analysis was then performed for bisphenol S, which showed significant association with asthma in the univariate analysis. Simple logistic regression model was done for univariate analysis (model 1). Multiple logistic regression model adjusted with confounders was performed, using (1) variables statistically related to asthma (*p* < 0.25) (model 2) and (3) all bisphenols and other variables theoretically related to asthma according to previous literature, as confounders (model 3). The outcome variables of the logistic regression were (1) asthma ever diagnosed (vs. never diagnosed), and (2) asthma diagnosed after 60 months (vs. never diagnosed or asthma diagnosed before 60 months. Next, multinomial logistic regression analysis was performed. For the outcome variable, participants were divided into three groups: those who had never been diagnosed with asthma, diagnosed before 60 months of age, and diagnosed after the age of 60 months. Since using spirometry was not possible before 60 months, we separately analyzed asthma diagnosed before and after 60 months of age [25]. Age and income were treated as categorical variables.

All analyses were performed considering the cluster, strata, and weights considered during sampling. The analysis was performed in R 3.6.3. The packages ‘survey’, ‘svyVGAM’, and ‘jtools’ were used for weighted survey data analysis.

## 3. Results

The general characteristics of the study participants and univariate analysis of asthma and other variables are listed in Table 1. In males, there were no significant differences in general characteristics and urinary bisphenols according to asthma diagnosis.

Among females, the median urinary BPS in the “ever diagnosed with asthma” group (median: 0.11 μg/g creatinine, 25th percentile: 0.01 μg/g creatinine, 75th percentile: 0.20 μg/g creatinine) was significantly higher than “never diagnosed with asthma” group (median: 0.03 μg/g creatinine, 25th percentile: 0.01 μg/g creatinine, 75th percentile: 0.08 μg/g creatinine) (*p* < 0.01) in univariate analysis. Serum IgE levels were also significantly higher in the “ever diagnosed with asthma” group than the “never diagnosed with asthma” group among females (*p* < 0.001).

**Figure 1:**
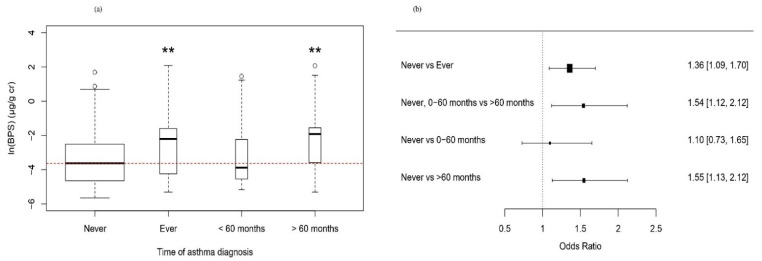
(**a**) Box and whisker plot of urinary bisphenol S and time of asthma diagnosis. Compared to the participants without diagnosed asthma, asthma diagnosed ever group, and asthma diagnosed >60 months of age shows higher median value, and statistically significant in univariate analysis using the Mann–Whitney U test, whereby ** *p* < 0.01. (**b**) Forest plot shows adjusted odds ratio and 95% confidence interval, which represent the change of odds ratio of categorical dependent variables per 1 unit of the increased/decreased independent variable: ln (urinary bisphenol S (µg/g creatinine)).

The median of BPS was 0.02 μg/g creatinine (25th percentile 0.01 μg/g creatinine, 75th percentile 0.11 μg/g creatinine) in the “asthma diagnosed before 60 months” group, and 0.15 μg/g creatinine (25th percentile 0.03 μg/g creatinine, 75th percentile 0.21 μg/g creatinine) in the “asthma diagnosed after 60 months” group. The median of BPS was significantly higher in the “asthma diagnosed after 60 months” group than in the “never diagnosed” group (*p* < 0.01). However, the median BPS showed no significant difference among the “never diagnosed with asthma” group and “diagnosed before 60 months” group (Figure 1a).

Correlations between the serum IgE and other continuous variables are presented in Table 2. Correlations among BPS, BPA, and BPF were significant in both sexes. Serum IgE levels did not show significant relationships with BPA, BPS, or BPF in either sex.

Results of multivariable analysis were presented in Table 3. Simple and multiple logistic regression analysis was performed for BPS, which was significantly related to asthma in univariate analysis among three urinary bisphenols. Model 1 is univariate analysis. For model 2, age, BMI, and BPA, which are potentially relevant variables in the univariate analysis, were adjusted. In model 3, income [26], cotinine [27], and BPF [14], which were reported to affect the diagnosis and prevalence of asthma from previous literatures, were additionally adjusted. BPS showed a significant positive association with lifetime diagnosis of asthma in all models. The increase in urinary BPS was associated with an increased odds ratio (OR) of “asthma diagnosed after 60 months” compared to “asthma diagnosed before 60 months” or “never diagnosed”. In multinomial logistic regression, according to the time of asthma diagnosis, there was no significant association in BPS between “never diagnosed” and “asthma diagnosed before 60 months” of age groups (*p* = 0.69). The increase of urinary BPS was associated with an increased OR of “asthma diagnosed after 60 months” compared to “never diagnosed” group (*p* < 0.01) (Figure 1b). For a lifetime diagnosis of asthma, BPS showed a significant correlation in all the models. The increase in BPS was associated with an increased OR of asthma diagnosed after 60 months. In multinomial logistic regression according to the time of asthma diagnosis, there was no significant association in BPS between the no asthma and the asthma diagnosed before 60 months groups (*p* < 0.69), but there was a significant relationship with the group diagnosed with asthma after 60 months compared to the no asthma group (*p* < 0.01).

## 4. Discussion

Bisphenol analogues disrupt endocrine secretion by acting similar to estrogen [20]. BPA has been known for its toxicity in the past, and its use is now regulated worldwide. The use and exposure of alternative substances, such as BPS and BPF, is increasing [28]. BPS and BPF also have chemical structures and properties similar to those of BPA; therefore, they are expected to be toxic to the human body. However, the relationship between allergic diseases in humans, such as asthma, and bisphenol analogues has not yet been clearly established. In this study, urinary BPS in 12–17-year-old Korean adolescents showed a significant association with asthma diagnosis in females, and in particular, there was a significant relationship with asthma diagnosed at the age of >60 months. No significant relationship was observed in males. Serum IgE levels did not show a significant relationship with the bisphenols.

It is difficult to use diagnostic tools, such as spirometry, in patients under 60 months of age; therefore, a clinical diagnosis is usually performed by assessing the symptoms (wheezing, cough, breathlessness, etc.) and response to treatment. However, as there are various diseases with similar symptoms, the validity of asthma diagnosis varies depending on the evaluation method, and the sensitivity or specificity may drop below 30% [29]. The gold standard diagnostic tool for asthma, such as spirometry and bronchodilator challenge, can be applied in patients over 6 years of age [25], so the diagnosis is more accurate. Therefore, we analyzed asthma diagnosed before and after 60 months of age. The results of this study, in which the association between BPS and asthma after 60 months was observed, showed the relationship between asthma and BPS more clearly.

BPS has been reported to be associated with interleukin (IL)-10, vascular endothelial growth factor, macrophage inflammatory protein 1b, IL-8, IL-1RA, and interferon gamma in in vitro studies [30]. In animal studies, BPS was associated with cytokines related to allergic reactions, such as IL-6, IL12a, and interferon c [31]. It is interesting to note that this association shows gender dimorphism. Estrogen is a hormone that affects the formation and function of T cells. T cells contain ER alpha and beta and are affected by estrogen [32]. Estrogen affects Th1 and Th2 cells and appears to induce Th1 responses at low doses and Th2 responses at high doses [33,34]. Bisphenols act as xenoestrogens; therefore, they can also undergo immunologic reactions similar to the action of estrogen [35]. An animal study showed that BPA, BPS, and BPF affect the modulation of the immune system through the estrogen receptor ER-α [36]. In particular, in an in vitro study, the genomic estrogenic activity of BPS was reported to be affected by the ERβ2 pathway, unlike BPA, in which ERα is the main pathway.

This study was conducted on 12–17-year-olds, and only female showed an association between asthma and BPS. The sexual dimorphism of the relationship between bisphenols, a xenoestrogen, and asthma has been an interesting study topic, but the direction is not consistent. A previous study analyzing the National Health and Nutrition Examination Survey of the United States showed that BPS and asthma were associated only in the male population aged 12 years and older [14]. One epidemiological study of BPA showed that asthma was associated with only males aged 5–17 years [15]. Another epidemiologic study of BPA and asthma showed that, across all ages, BPA was associated with asthma with elevated eosinophil and IgE levels in women only, but not in men [16]. In animal experiments, complex dose- and sex-specific effects of BPA on the cellular and microanatomical structures of the spleen, which controls the immune system, were observed [37]. In another animal study performed on mice, maternal BPA was not associated with asthma development at 10 times the no-observed-adverse-effect-level during pregnancy and lactation, but lifetime exposure increased asthma risk according to the period of BPA exposure. However, exposure to BPA during the sensitization period reduces asthma manifestations [38]. At higher concentrations, maternal bisphenol A increased IgE levels and eosinophilic infiltration in the lungs during offspring sensitization [39]. Bisphenols act on receptors of various organs because of the characteristics of endocrine disruptors and have a non-monotonic dose–response curve depending on the dose [40]. The various toxic effects according to the dose, sex, exposure period, and type of analogues of bisphenols shown in animal experiments under controlled conditions would be associated with the diverse results of various epidemiological studies with relatively few controlled factors. Although it is difficult to clearly identify the exposure and toxicity outcomes of toxic substances that are ubiquitous in the environment, such as bisphenols in human studies, a detailed analysis considering the dimorphism of gender, age, exposure period, etc., will be required for designing future studies in the human body.

As the most critical limitation of this study, a cross-sectional study was performed to estimate the exposure of bisphenols in the body through a single time-point sample of urinary BPS, and this was compared with the prevalence of asthma in the past. The timing of asthma diagnosis could not be precisely specified, and the prevalence was compared with that of biomarkers after onset. In addition, urinary BPS better reflects acute exposure rather than chronic [41]. There is still uncertainty as to whether urinary BPS is valid as an exposure index because of the lack of historical exposure data or data on toxicokinetics and toxicodynamics. However, in studies on BPA, there is evidence that although it decreases significantly in a short period of time, the entire amount does not escape and accumulates in fat [42]. Bisphenols have been identified in fat in animals, and they are likely to accumulate in fat in the human body and be released through urine [43,44]. Urinary BPA levels have been reported to reflect long-term BPA exposure [45]. Although studies on urinary BPS as an exposure biomarker are lacking, BPS is more stable than BPA and persists longer in the body [17,18]. Thus, it is thought to reflect chronic exposure, such as urinary BPA. Therefore, we regarded urinary BPS as an index that reflects the lifetime BPS exposure propensity. Although it is not possible to identify the causal relationship between BPS and asthma in this cross-sectional study, considering the pathophysiological evidence revealed by previous animal and in vitro studies, this study suggests the possibility that BPS will affect the onset of asthma.

Another limitation of this study is that the diagnosis of asthma does not utilize diagnostic tools such as spirometry or medical records. However, the lifetime prevalence of asthma among the subjects, who were 12–17 years old, was 11.5% for men and 7.8% for women. Previous studies reported the prevalence of asthma in the Republic of Korea at the level of 5.48–7.86% (2002–2015, under the age of 18 years) [46] and 11.1–16.5% (2010–2014, under the age of 19 years) [47] (analyzed using medical records). It is expected that there will be no critical difference in the actual prevalence of asthma diagnosed by a doctor. Another limitation is that our study results are based on data from the KoNEHS and reflect the Korean population well, but Koreans nearly comprise a single ethnic group consisting of East Asians [48]. Considering that allergic diseases are affected by race and genetics [49], it is difficult to extrapolate the results of this study to other races and countries. However, it is also a strength that the results reflect the Korean population. This study is a secondary data analysis study using the KoNEHS data. Among the study subjects in the population, there were few samples of treated asthma patients with current asthma symptoms, and laboratory data related to asthma disease entities such as eosinophils and spirometry are not available and could not be analyzed.

## 5. Conclusions

In this study, a relationship between asthma, especially its onset after 60 months of age, and BPS was observed in female Korean adolescents. Although causality was not confirmed, the results of this study suggest that BPS, like BPA, can affect human immunity. It also suggests that BPS may have more severe toxic effects in certain sexes and age groups. In the future, the toxicity of BPS beyond asthma onset, such as allergic disease and humoral immunity, should be elucidated through additional longitudinal and mechanistic studies.

## Figures and Tables

**Table 1:** General characteristics of study participants.

Sex	Male (*N* = 478)			Female (*N* = 422)		
Asthma	Ever Diagnosed (*N* = 55)	Never Diagnosed (*N* = 423)	*p*-Value	Ever Diagnosed (*N* = 33)	Never Diagnosed (*N* = 389)	*p*-Value
Age (years)			0.45			0.15
12	4 (8.1%)	70 (16.4%)		3 (9.2%)	64 (16.4%)	
13	7 (12.2%)	69 (16.3%)		4 (12.2%)	53 (13.6%)	
14	7 (12.4%)	61 (14.4%)		3 (10.5%)	63 (16.1%)	
15	10 (17.9%)	76 (18%)		14 (41.4%)	74 (19.1%)	
16	14 (24.9%)	76 (17.8%)		3 (10.5%)	77 (19.8%)	
17	13 (24.5%)	72 (17.1%)		5 (16.1%)	58 (14.9%)	
Family income (per month)			0.25			0.81
<1 million won	2 (3.1%)	9 (2.2%)		1 (2.5%)	10 (2.5%)	
1–2 million won	2 (4.3%)	46 (10.8%)		5 (15.2%)	42 (10.8%)	
2–3 million won	6 (10.1%)	74 (17.4%)		6 (19.6%)	51 (13.2%)	
3–5 million won	13 (23.1%)	120 (28.3%)		7 (22.5%)	132 (33.9%)	
5–7 million won	13 (23.9%)	92 (21.8%)		7 (20.7%)	94 (24.2%)	
≥million won	11 (19.6%)	57 (13.5%)		5 (16.1%)	39 (9.9%)	
Unknown	9 (16.0%)	26 (6.1%)		1 (3.4%)	21 (5.5%)	
BMI (kg/m^2^)	21.3 (19.7, 24.1)	22.2 (19.8, 24.9)	0.44	22.2 (20.5, 24.8)	21.0 (19.5, 22.8)	0.08
Urinary BPA (μg/g creatinine)	0.67 (0.37, 0.67)	0.86 (0.46, 1.56)	0.33	1.37 (0.57, 2.31)	1.03 (0.55, 1.85)	0.22
Urinary BPS (μg/g creatinine)	0.02 (0.01, 0.08)	0.03 (0.01, 0.07)	0.67	0.11(0.01, 0.20)	0.03 (0.01, 0.08)	<0.01
Urinary BPF (μg/g creatinine)	0.04 (0.03, 0.11)	0.04 (0.03, 0.11)	0.65	0.08 (0.04, 0.17)	0.05 (0.03, 0.15)	0.40
Urinary cotinine (μg/g creatinine)	1.57 (1.05, 12.40)	1.57 (1.05, 2.43)	0.32	1.29 (0.85, 1.92)	1.30 (0.82, 2.78)	0.95
Time of the first asthma diagnosis						
≤60 months	29 (52.4%)	-	-	17 (49.9%)	-	-
>60 months	26 (47.6%)	-	-	17 (50.1%)	-	-
Current Asthma Treatment						
Yes	3 (5.4%)	-	-	1 (3.9%)	-	-
No	52 (94.6%)	-	-	32 (96.1%)	-	-
Serum IgE (IU/mL)	249.6 (91.6, 539.2)	98.8 (25.1, 213.2)	0.06	152.7 (85.2, 544.0)	127.3 (48.6, 330.0)	<0.001

BMI: body mass index, BPA: bisphenol A, BPF: bisphenol F, BPS: bisphenol S. Sampling weight was applied for all values and analysis and rounded. *p*-value was calculated using the Mann–Whitney U test for continuous variables, and chi square test for categorical variables. Five missing values of blood IgE among female participants: 1 from asthma diagnosed group and 4 from never diagnosed group.

**Table 2:** Correlation analysis between serum IgE, urinary bisphenol analogues, and possible confounders.

		ln (Urinary BPS)	ln (Urinary BPA)	ln (Urinary BPF)	Age	ln (Urinary Cotinine)	BMI
ln (serum IgE)	Female	0.01	0.00	−0.01	0.05	−0.01	0.11 *
	Male	−0.01	0.02	0.02	−0.05	−0.07	0.12 *

* *p* < 0.05. Correlation coefficient was calculated by Pearson’s correlation method. Variables with right-skewed distribution were log-transformed. *p*-values were calculated using non-parametric bootstrap method. Resampling was performed 2000 times.

**Table 3:** Multivariable analysis between asthma diagnosis and urinary bisphenol S.

		Asthma (Never vs. Ever Diagnosed) *				Asthma (Never + Diagnosed ≤ 60 Months vs. Diagnosed > 60 Months) **				Asthma (Never vs. Diagnosed ≤ 60 Months vs. Diagnosed after > 60 Months) ***			
			OR	95% CI	*p*-Value		OR	95% CI	*p*-Value		OR	95% CI	*p*-Value
ln (urinary BPS) (μg/g creatinine)	Model1	Asthma ever	1.49	(1.20–1.86)	<0.001	Asthma (>60 mo)	1.72	(1.33–2.23)	<0.001	Asthma (≤60 mo)	1.26	(0.77–2.07)	0.36
										Asthma (>60 mo)	1.75	(1.37–2.22)	<0.001
	Model2	Asthma ever	1.37	(1.09–1.73)	<0.05	Asthma (>60 mo)	1.51	(1.26–1.81)	<0.001	Asthma (≤60 mo)	1.18	(0.73–1.9)	0.51
										Asthma (>60 mo)	1.53	(1.29–1.81)	<0.001
	Model3	Asthma ever	1.36	(1.09–1.70)	<0.05	Asthma (>60 mo)	1.54	(1.12–2.12)	<0.05	Asthma (≤60 mo)	1.10	(0.70–1.58)	0.69
										Asthma (>60 mo)	1.55	(1.13–2.12)	<0.01

Odds ratio (OR) and 95% confidence interval represent the change of OR of categorical dependent variables per 1 unit of the increased/decreased independent variable: ln (bisphenol S) (μg/g creatinine). Multiple logistic regression and multinomial regression were performed to calculated OR and 95% CIs. Model 1 is univariate analysis. Model 2 was adjusted by age, BMI, and ln (urinary BPA). Model 3 was adjusted by age, BMI, ln (urinary BPA), ln (urinary BPF), ln (urinary cotinine), and family income. Age and family income were applied as categorical variables. Reference: * Never diagnosed, ** Never diagnosed + diagnosed ≤60 months, *** Never diagnosed.

## Data Availability

This study used data from the third Korean National Environmental Health Survey 2016 data, which is open to any researcher after request.
